# SBLC: a hybrid model for disease named entity recognition based on semantic bidirectional LSTMs and conditional random fields

**DOI:** 10.1186/s12911-018-0690-y

**Published:** 2018-12-07

**Authors:** Kai Xu, Zhanfan Zhou, Tao Gong, Tianyong Hao, Wenyin Liu

**Affiliations:** 1 School of Computer Science and Technology, Guangdong University of Technology, Guangzhou, China; 2 School of Information Science and Technology, Guangdong Universities of Foreign Studies, Guangzhou, China; 3 Educational Testing Service, Princeton, NJ USA; 4 Center for Linguistics and Applied Linguistics, Guangdong University of Foreign Studies, Guangzhou, China; 5 School of Computer Science, South China Normal University, Guangzhou, China

**Keywords:** Biomedical informatics, Text mining, Machine learning, Neural networks

## Abstract

**Background:**

Disease named entity recognition (NER) is a fundamental step in information processing of medical texts. However, disease NER involves complex issues such as descriptive modifiers in actual practice. The accurate identification of disease NER is a still an open and essential research problem in medical information extraction and text mining tasks.

**Methods:**

A hybrid model named Semantics Bidirectional LSTM and CRF (SBLC) for disease named entity recognition task is proposed. The model leverages word embeddings, Bidirectional Long Short Term Memory networks and Conditional Random Fields. A publically available NCBI disease dataset is applied to evaluate the model through comparing with nine state-of-the-art baseline methods including cTAKES, MetaMap, DNorm, C-Bi-LSTM-CRF, TaggerOne and DNER.

**Results:**

The results show that the SBLC model achieves an F1 score of 0.862 and outperforms the other methods. In addition, the model does not rely on external domain dictionaries, thus it can be more conveniently applied in many aspects of medical text processing.

**Conclusions:**

According to performance comparison, the proposed SBLC model achieved the best performance, demonstrating its effectiveness in disease named entity recognition.

## Background

Medical named entities are prevalent in biomedical texts, and they play critical roles in boosting scientific discovery and facilitating information access [1]. As a typical category of medical named entities, disease names are widely used in biomedical studies [2], including disease cause exploration, disease relationship analysis, clinical diagnosis, disease prevention and treatment [3]. Major research tasks in biomedical information extraction depend on accurate disease named entity recognition (NER) [4–8], and how to accurately identify disease named entities is a fundamental and essential research problem in medical information extraction and text mining tasks.

Disease NER involves many complex issues, which induce difficulties in actual practice [3]. Disease names are usually generated by combining Greek and Latin roots and affixes, e.g., *hemo-chromatosis*. More and more unknown names are difficult to identify from a morphology aspect. Many disease names also frequently contain disease descriptive modifiers, e.g., *liver cancer*. These modifiers may be related to human body parts or degrees of disease, e.g. *recurrent cat-eye syndrome*. This may cause difficulties in identifying modifiers from other types of medical named entities (e.g., *syndrome*). Moreover, disease names may have multiple representation forms. For instance, *hectical complaint* and *recurrent fever* are the same disease but represented differently. Finally, there exist a large amount of disease name abbreviations in medical texts. Some of them may not be standard, such as those user-defined abbreviations listed in the appendix of clinical trial texts.

There are large number of biomedical texts, e.g., PubMed, PMC OA full texts, and Wikipedia. In order to effectively obtain the semantic information from the texts, word embedding training method named Negative Sampling (NEG) Skip-gram [9] was proposed by Mikolov et al. to learn high quality vector representations from a large number of unstructured texts. This method could speed up the vector training process and generate better word embeddings. The method simplified the traditional neural network structure, and thus could adapt to a large number of texts. It could also automatically generate semantic representations of words in text context. Recently, many deep neural networks, such as the Long Short Term Memory network (LSTM) model [10], have been widely used to extract text context features. A variety of relevant models that integrate LSTM to train word contextual features and Conditional Random Field (CRF)-based methods to optimize word sequence parameters have been widely used in NER tasks [11]. These models improved the feature extraction process by reducing the work-load of feature selection. In addition, word embeddings have been proved to be effective in NER tasks [12]. Motivated by both the effectively applied LSTM model and the usefulness of word embeddings, this paper combines the word embeddings containing the semantics of disease named entities with LSTM to improve the performance of disease NER tasks.

To this purpose, we propose a new model named SBLC for disease NER. The model is based on word embeddings, bidirectional LSTM and CRF. As a multi-layer neural network, the model consists of three layers. The first layer is word embedding, which is generated from medical resources through massive medical text training. The second layer is Bi-LSTM, which is used to obtain the context of semantic structures. The third layer is CRF, which captures relationship among token labels. We evaluate the SBLC model by comparing it with the state-of-the-art methods including NCBI, UMLS, CMT, MeSH, cTAKES, DNorm and TaggerOne. Based on the standard publicly available NCBI disease dataset that contains 6892 disease named entities, the SBLC model achieves an F1 score of 0.862, outperforming all the other baseline methods.

The major contributions of this paper lie in the following two aspects. First, the proposed SBLC model systematically combines word embedding, bidirectional LSTM and CRF for disease NER tasks. Second, this revised model by integrating Ab3P improves the current performance compared with state-of-the-art methods on a publically available dataset.

The rest of the paper is organized as follows: The section Related Work gives a brief overview of the background of the disease NER and related work. The section Methods introduces the methodology of the SBLC model. The section Result presents the evaluation of the proposed SBLC model. The section Discussion analyzes error cases, discusses properties of medical semantic words, and points out the limitations of our model. Finally, the section Conclusion concludes this study.

## Related work

### Disease NER

In medical domain, most existing studies on disease NER mainly used machine learning methods with supervised, unsupervised or semi-supervised training. For example, Dogan et al. [2] proposed an inference-based method which linked disease names mentioned in medical texts with their corresponding medical lexical entries. The method, for the first time, used Unified Medical Language System (UMLS) [13] developed by the National Library of Medicine in the NCBI disease corpus. Some similar systems, such as MetaMap [14], cTAKES [15], MedLEE [16], SymText / MPlus [17], KnowledgeMap [18], HiTEX [19] have been developed utilizing UMLS. Although UMLS could cover a wide range of medical mentions, many of these methods failed to identify disease mentions not appearing in the UMLS. In addition, the NER efficiency in terms of accuracy was not sufficiently high for practical usage. For example, the F1 in NCBI dataset of official MetaMap was only 0.559 as reported in [2].

DNorm [3] was one of the recent studies using a NCBI disease corpus and a MEDICS vocabulary. It combined MeSH [20] and OMIM [21]. DNorm learned the similarity between disease names directly from training data, which was based on the technology of paired learning to rank (pLTR) strings normalization. Instead of solely relying on medical lexical resources, DNorm adopted a machine learning approach including pattern matching, dictionary searching, heuristic rules. By defining a vector space, it converted disease mentions and concepts into vectors. DNorm achieved an F1 score of 0.809 on the NCBI disease corpus.

In 2016, Leaman and Lu proposed the TaggerOne [22]. It was a joint model that combined NER and normalized machine learning during training and predicting to overcome the cascading error of DNorm. TaggerOne consisted of a semi-Markov structured linear classifier for NER and a supervised semantic index for normalization, and ensured high throughput. Based on the same NCBI disease corpus, TaggerOne achieved an F1 score of 0.829.

With respect to the methods applying deep learning to NER, some neural network models that could automatically extract word representation characteristics from raw texts have been widely used in the NER field (e.g., [23]). Using deep learning, some sequence annotation methods were also proposed and applied to disease NER tasks (e.g., [24, 25]). As a typical method, Pyysalo et al. [12] used word2vec to train a list of medical resources, and obtained a better performance on a NCBI Disease corpus. Recently, Wei et al. proposed a multi-layer neural network, DNER [24], which used GENIA Tagger [26] to extract a number of word features including words, part-of-speech tags, words chunking information, glyphs, morphological features, word embeddings, and so on. After extraction, the word features were embedded as inputs to a bidirectional Recurrent Neural Network model, and other features like POS tags were used for a CRF model. The normalization method of dictionary matching and the vector space model (VSM) were used together to generate optimized outputs. The overall performance of the model in terms of F1 score was 0.843 on the NCBI disease corpus. To our knowledge, DNER was the best performance deep learning-based method.

Motivated by the benefits of word embedding and deep learning from the existing research, we intend to utilize external medical resources for word representation and combine bidirectional LSTM and CRF for NER recognition. We use a large number of medical resources to train the word embeddings model in an unsupervised manner, and combine the deep learning techniques for disease NER tasks.

### Word embedding training

Success of machine learning algorithms usually depended on appropriate data representation, since different representations could capture different features of the data. Distributed word representation proposed by Hinton [27], has been widely used. The word distribution hypothesis held that the words in a similar context have similar meanings, which convey similarities in semantic dimensions. Along with the recent development of machine learning techniques, more and more complex models have been trained on larger datasets and achieved superior performance [28].

Mikolov et al. [29] proposed a skip-gram method for calculating vector representations of words in large data sets. The compositions of disease named entities often contained rare medical words. In order to improve the computational efficiency, the Skip-gram model removed the hidden layer so that all words in input layer shared a mapping layer. In the skip-gram method, Negative Sampling (NEG) was used. It was a simplified version of Noise Contrastive Estimation (NCE) [30]. NEG simplified NCE by guaranteeing word vector quality and improving training speed. NEG no longer used a relatively complex Huffman tree, but rather a relatively simple random negative sample, which could be used as an alternative for hierarchical softmax.

Motivated by the related work, particularly from Mikolov et al. [9, 29], we apply the NEG skip-gram method for disease NER. The method is described as follows. Given a training text sequence *w*_1_, …, *w*_*T*_, at position *t*, the distribution score *s*(*w*, *c*; *θ*) for the true probability model was calculated using Eq. (1). The target of *w* was a set of context words *w*_*t* − *n*_, …, *w*_*t* − 1_, *w*_*t* + 1_, …, *w*_*t* + *n*_.

1 $$ s\left({w}_t,{c}_t;\theta \right)={v}_{w_t}^T{v}_{w_{t+j}}^{\prime },-n\le j\le n,j\ne 0 $$

When using the negative sampling method, *k* negative cases (${\tilde{w}}_{t,i},1\le i\le k$) were randomly sampled in the noise distribution *Q*(*w*) for each positive case (*w*_*t*_, *c*_*t*_). *σ* was a logistic function. The negative function for negative samples was shown in Eq. (2):

2 $$ {\displaystyle \begin{array}{l}{L}_{\theta}\left({w}_t,{c}_t\right)=\log P\left(y=1|{w}_t,{c}_t\right)+\sum \limits_{i=1}^k\log \left(1-P\left(y=1|{\tilde{w}}_{t,i},{c}_t\right)\right)\\ {}\kern3.75em =\log \sigma \left(s\left({w}_t,{c}_t;\theta \right)\right)+\sum \limits_{i=1}^k\log \sigma \left(-s\left({\tilde{w}}_{t,i},{c}_t;\theta \right)\right)\end{array}} $$

The value *k* was determined by the size of the data. Normally, *k* ranged within [5, 20] in a small-scale data, while decreased to [2, 5] in a large-scale data [9]. Equation (2) could be solved by a random gradient rise method.

### Bi-LSTM & CRF

As a typical deep learning method, the long and short memory network (LSTM) [10] was usually used for annotation tasks of text sequences. LSTM, as shown in Eq. (3), could capture long distance information by adding several threshold cells which controlled the contribution of each memory cell. Therefore, LSTM enhanced the ability of keeping long distance context information. Longer contextual information could help the model to learn semantics more precisely.

3 $$ {\displaystyle \begin{array}{l}{i}_t=\sigma \left({W}_{xi}{x}_t+{W}_{hi}{h}_{t-1}+{W}_{ci}{c}_{t-1}+{b}_i\right)\\ {}{c}_t=\left(1-{i}_t\right)\odot {c}_{t-1}+{i}_t\odot \tanh \left({W}_{xc}{x}_t+{W}_{hc}{h}_{t-1}+{b}_c\right)\\ {}{o}_t=\sigma \left({W}_{xo}{x}_t+{W}_{ho}{h}_{t-1}+{W}_{co}{c}_t+{b}_o\right)\\ {}{h}_t={o}_t\odot \tanh \left({c}_t\right)\end{array}} $$

Bidirectional LSTM (Bi-LSTM) could simultaneously learn forward and backward information of input sentences and enhance the ability of entity classification. A sentence *X* containing multiple words could be represented as a set of dimension vectors (*x*_1_, *x*_2_, …, *x*_*n*_).${\overrightarrow{y}}_t$ denoted the forward LSTM and ${\overleftarrow{y}}_t$ denotes the backward LSTM. ${\overrightarrow{y}}_t$ and ${\overleftarrow{y}}_t$ were calculated by capturing from the LSTM the preceding and following information of the word *t*, respectively. The overall representation was achieved by generating the same backend sequence in LSTM. This pair of forward and backward LSTMs was Bi-LSTM. This representation preserved the context information for the word *t*.

Since there was more and more research focusing on Bi-LSTM and Conditional Random Field (CRF) in NER tasks, the following of this subsection described CRF. It was first introduced as a sequence data tag recognition model by Lafferty et al. [11]. Considering that the target of NER was label sequences, linear chain CRF could compute the global optimal sequence, thus it was widely used to solve NER problems. The objective function of a linear chain CRF was the conditional probability of the state sequence *y* given the input sequence *x, as* shown in Eq. (4).

4 $$ P\left(y|x\right)=\frac{1}{z(x)}\exp \left(\sum \limits_{k=1}^K{\lambda}_k{f}_k\left({y}_t,{y}_{t-1},{x}_t\right)\right) $$

*f*_*k*_(*y*_*t*_, *y*_*t* − 1_, *x*_*t*_) was a characteristic function. *λ*_*k*_ denoted the learning weights of the function features, while *y*_*t* − 1_ and *y*_*t*_referred to the previous and the current states, respectively. *Z*(*x*) was the normalization factor for all state sequences, as shown in Eq. (5).

5 $$ Z(x)=\sum \limits_y\exp \left(\sum \limits_{k=1}^K{\lambda}_k{f}_k\left({y}_t,{y}_{t-1},{x}_t\right)\right) $$

The maximum likelihood method and numerical optimization L-BFGS algorithm were used to solve the parameter vector $\overrightarrow{\lambda}=\left\{{\lambda}_1,\dots, {\lambda}_k\right\}$ in training process. The viterbi algorithm was used to find the most likely hidden state sequences from observed sequences [31].

## Methods

**Fig. 1:**
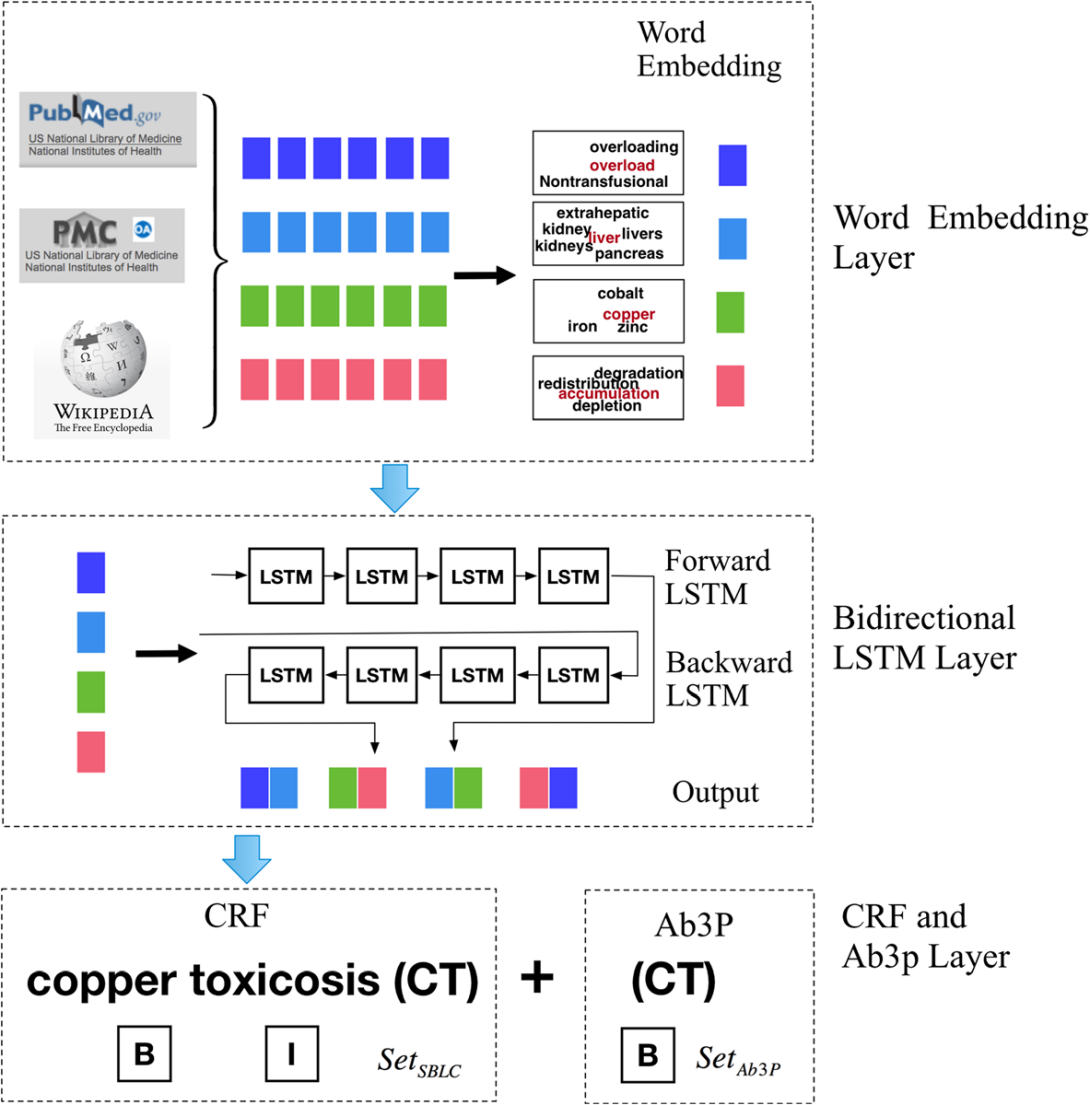
The overall architecture of the proposed SBLC model including three layers: The first layer is word embedding containing word embeddings trained on three large-scale datasets. The second layer is Bi-LSTM used to learn context information. The third layer is CRF and Ab3p capturing the relationship among word part-of-speech labels

This paper presents a new model SBLC for disease named entity recognition based on semantic word embedding, bidirectional LSTM, and CRF. The model consists of three layers: 1) a semantic word embedding layer, 2) a bidirectional LSTM layer, and 3) a CRF and Ab3p layer. The overall architecture of the SBLC model shown in Fig. 1.

In the model, we first train semantic word vectors on three corpora including PubMed, PMC OA full text and Wikipedia. The trained word vectors are then projected to the vectors trained on a standard NCBI corpus. The word vectors containing text semantic information are input to the Bi-LSTM layer. The NCBI training corpus is further used for Bi-LSTM parameter training. We optimize sequence parameters by the CRF layer. Finally, the model identifies disease abbreviations using an Ab3P module.

The first layer is word embedding. The Skip-gram model based on Negative Sampling is used to train word embeddings on the three large-scale medical datasets. Based on a previous work [12], we extract the texts from PubMed, PMC Open Access (OA), and Wikipedia. A total of 22,120,000 abstract records from PubMed, 672,000 full-texts from PMC OA, and 3,750,000 articles from Wikipedia are retrieved by the end of 2013. The finally extracted texts as a corpus contain a total of 5.5 billion words. The corpus is then used as the training dataset for word embedding generation.

The second layer is Bi-LSTM, which is used to learn context information. LSTM captures long distance information through a threshold unit, thus it can learn more semantic features through longer contextual information. Using the Bi-LSTM structure can simultaneously learn the context information of preceding and following sentences. From our previous empirical studies, the Bi-LSTM can enhance entity classification performance.

The third layer is CRF and Ab3p, which captures the relationship among word part-of-speech labels. We use NLTK toolkit [32], a widely used natural language processing tool, for part-of-speech labeling. In the CRF, the Viterbi algorithm is used to solve the global optimal sequence problem. Finally, the BIO method is used for NER annotation and the Ab3P is used to identify additional disease abbreviations.

In general, a disease NER task can be regarded as a process of assigning named entity tags to words. A single named entity may consist of multiple words in order. Accordingly, we use the BIO method for sequenced-word labeling. Each word is marked with BIO labels. A word is tagged with a *B* label if it is at the beginning of a named entity. If the word is inside the entity but not at the beginning, it is tagged as *I*. Words that are not named entities are marked as *O*.

The labels of named entities are mutually dependent. For example, an *I*-PERSON cannot appear after a *B*-LOCATION label. Therefore, the BIO labels cannot be tagged independently. We use a CRF method to calculate the possibility score of each label from the Bi-LSTM output. The objective function *s*(*X*,*y*), as shown in Eq. (6), is used to calculate the probability of each label. The higher the value, the higher probability of the predicted label to be chosen.

6 $$ s\left(X,y\right)=\sum \limits_{i=1}^n{P^{sem}}_{i,{y}_i}+\sum \limits_{i=0}^n{A}_{y_i,{y}_{i+1}} $$

For an input sentence set *X* = (*x*_1_, *x*_2_, …, *x*_*n*_), *P*^*sem*^ is a score matrix, which is the output of the bidirectional LSTM network containing the medical semantic features. *P*^*sem*^ is of size *n* × *k*, where *k* is the number of different BIO labels and it is set to 3 in this paper. *A* is a matrix of transition scores and *A*_*i*, *j*_ represents the transition score from the BIO *label*_*i*_ to *label*_*j*_. *y*_0_ and *y*_*n*_ are the beginning and ending labels of a sentence, respectively.

We use a softmax function *p*(*y*|*X*) to calculate the probability of sequence *y* from all possible label sequences, as shown in Eq. (7)*.*

7 $$ p\left(y|X\right)=\frac{\exp \left(s\left(X,y\right)\right)}{\sum_{\tilde{y}\in {Y}_X}\exp \left(s\left(X,\tilde{y}\right)\right)} $$

The final computation task is to find the point estimate y* of all possible outputs *y* such that the conditional log-likelihood probability *P*(*y|X*) is maximized, as shown in Eq. (8).

8 $$ {y}^{\ast }=\arg \max \left(\log P\left(y|X\right)\right) $$

In the task of disease NER, disease abbreviations are often interfered by other non-disease abbreviations. For example, a disease name CT appearing in a clinical text may refer to Computed Tomography (non-disease) or Copper Toxicosis (Wilson disease). Thus, the identification of CT as Computed Tomography is incorrect.

The abbreviation recognition is not effective using solely word embeddings generated by the NEG skip-gram training, since the disease abbreviations are easily conflicted with other types of non-disease abbreviations. Taking the same example, CT is expected to be classified as Copper Toxicosis (ID 215600 in OMIM (Online Mendelian Inheritance in Man)). However, the most similar vocabularies associated with the word embeddings are the following 5 ranked tuples (noncontrast CT, 0.8745), (MDCT ray, 0.8664), (Computed tomography, 0.8643), (non-contrast, 0.8621), and (unenhanced, 0.8505), where the first tuple element refers to the words relevant to CT and the second element is their similarity values. However, the similarity between CT and target word Copper Toxicosis is as low as 0.003, causing the difficulty in the identification of disease abbreviation Copper Toxicosis. To that end, we use Ab3P [33], available at http://www.ncbi.nlm.nih.gov/CBBresearch/Wilbur/, to identify disease abbreviations. Evident in previously reported results, Ab3P has an F1 score of 0.9 and 0.894 ​​on the Medstract corpus and the MEDLINE annotation set, respectively. It defines short form (SF) as abbreviations and long form (LF) as the full representations of the abbreviations. Ab3P uses relaxed length restrictions and tried to find the best LF candidates by searching for the most reliable strategy out of seventeen strategies. For example, strategy FC denotes that a SF character matches the 1st character of a word in LF. Strategy FCG denotes that a SF character matches the character following a non-alphanumeric and non-space character in LF.

The BIO labels for the identified abbreviations by SBLC and Ab3P are *Set*_*SBLC*_ and *Set*_*Ab*3*P*_, respectively. The final label sets are computed as*Set*_*SBLC*_ ∪ *Set*_*Ab*3*P*_. If there is no identification output for an abbreviation using SBLC, the identified label by Ab3P is applied as the final result. In cases the identified labels from SBLC and Ab3P are different, the labels by Ab3P are taken as the correct identification. In this way, Ab3P in identifying abbreviations of disease named entities is used to supply the SBLC, thus improving the overall NER performance.

## Results

### Dataset

We use a publicly available dataset, the NCBI disease corpus [2], to evaluate the performance of the proposed SBLC model. The dataset is developed and annotated by the research groups from American National Center for Biotechnology Information (NCBI) and American National Institutes of Health (NIH). It has been frequently used in disease NER tasks [3, 22, 24]. The dataset contains 793 article abstracts from PubMed, and includes over 6000 sentences and 2136 unique disease concepts. The dataset is manually annotated by 14 persons having medical informatics research backgrounds and medical text annotation experiences. The dataset consists of three sub-datasets: a training data set (593 texts), a development data set (100 texts), and a test data set (100 texts). Detailed statistics information of the NCBI dataset is shown in Table 1.

**Table 1:** The statistics of the NCBI dataset for disease NER

Characteristics	Training	Developing	Testing	Total
# of PubMed article abstracts	593	100	100	793
# of annotated disease mentions	5145	787	960	6892
# of unique annotated disease mentions	1710	368	427	2136
Avg. sentences/abstract	10	10	10	10
Avg. words/sentence	20	22	22	21
Avg. words/abstract	217	226	232	225

### Baseline

To evaluate the effectiveness of the SBLC, the following 9 baseline methods are used in performance comparison:

1. Dictionary look-up method [2]. It uses Norm from the SPECIALIST lexical tools to identify disease names in the MEDIC lexicon.
2. cTAKES [15]. The cTAKES NER component implements a dictionary look-up algorithm within a noun-phrase look-up window. The dictionary is a subset of UMLS, including SNOMED CT and RxNORM concepts guided by extensive consultations with clinical researchers and practitioners. Each named entity is mapped to a concept from the terminology. The cTAKES is available at http://ctakes.apache.org/. In the comparison, we use the latest version cTAKES 4.0.
3. MetaMap [14]. MetaMap is based on lexical look-up to identify the UMLS Metathesaurus concepts in biomedical texts. In the experiment, we use MetaMap MEDIC filtering to restrict output results to disease names.
4. The Inference Method [2]. It tries to link diseases to their corresponding medical lexical entries. It designs string matching rule combinations that map annotated strings to standard disease dictionaries. The method was tested by the manually annotated AZDC disease corpus and the PubMed abstract texts.
5. DNorm [3]. The method is based on pairwise learning to rank (pLTR), which has been successfully applied to large optimization problems in information retrieval. It learns similarities between mentions and concept names, including synonymy and polysemy.
6. CRF + UMLS, CRF + CMT, CRF + MeSH [34]. These are several hybrid combination strategies involving CRF and UMLS, CRF and Convergent Medical Terminology (CMT), as well as CRF and Medical Subject Headings (MeSH).
7. C-Bi-LSTM-CRF [34]. It extracts the prefix and suffix information for each word at the character-level in training text. The method consists of three layers. The first layer is a character-based Bi-LSTM layer designed to learn character-level expressions of words. The second layer is a word-based Bi-LSTM layer. The third layer is a CRF layer, which captures the relations among labels.
8. TaggerOne [22]. This method is developed by the National Center for Biotechnology Information, USA. It uses a semi-Markov structured linear classifier for NER and normalization, simultaneously performs NER and normalization during training and prediction.
9. DNER [24]. Based on a deep learning method Bi-RNN, this method recognizes named entities using a support vector machine classifier. Dictionary matching and vector space model based normalization method are used to align the recognized mention-level disease named entities in MeSH.

We further analyze the functional characteristics of all the baseline methods in terms of using “dictionary look-up”, “disease name normalization”, “word embedding”, “LSTM”, and “CRF”, as shown in Table 2. “Y” means that a method contains a specific function and “N” means not. As can be seen in the table, most of the methods use disease name normalization approach and half of them use CRF. Only SBLC and C-Bi-LSTM-CRF use LSTM. SBLC is the only method that uses word embedding and it does not rely on dictionary look-up nor disease name normalization.

**Table 2:** Parameter combination comparison

Methods	Dictionary look-up	Disease name normalization	Word embedding	LSTM	CRF
Dictionary look-up [2]	Y	Y	N	N	N
cTAKES [15]	Y	Y	N	N	Y
MetaMap [14]	Y	Y	N	N	N
Inference Method [2]	Y	Y	N	N	N
CRF + UMLS [34]	Y	Y	N	N	Y
CRF + CMT [34]	Y	Y	N	N	Y
CRF + MeSH [34]	Y	Y	N	N	Y
DNorm [3]	Y	Y	N	N	N
C-Bi-LSTM-CRF [34]	N	N	N	Y	Y
TaggerOne [22]	N	Y	N	N	N
DNER [24]	N	Y	N	N	Y
SBLC	N	N	Y	Y	Y

### Evaluation metrics

We use three widely used evaluation metrics, precision, recall and F1-score, in disease NER studies [2, 3, 24, 34, 35] and other types of NER studies [23, 25, 31]. There are four possible outcomes for an instance in a testing data: An instance will be classified as a disease when it is truly a disease (true positive, TP); it will be classified as a disease when it is actually a non-disease (false positive, FP); it will be classified as a non-disease when it is actually a disease (false negative, FN); or it will be classified as a non-disease and it is truly a non-disease (true negative, TN). Based on these 4 possible outcomes, precision, recall and F1-score are defined as follows:

Precision: the proportion of instances that are correctly labeled as diseases among those labeled as diseases.

9 $$ Precision=\frac{TP}{TP+ FP} $$

Recall: the proportion of disease instances that are correctly labeled.

10 $$ Recall=\frac{TP}{TP+ FN} $$

F1 score: the harmonic mean of precision and recall.

11 $$ F 1=\frac{2\times Precision\times Recall}{Precision+ Recall} $$

### Parameter tuning

In SBLC, there are a number of parameters. In the parameter tuning process, we try different combinations of the parameters and record the corresponding performances in terms of F1 scores based on the training dataset. Eventually, we obtain a list of optimized parameter values, as shown in Table 3.

**Table 3:** The optimized parameter settings of the LSTM network

Parameter	Setting	Description
Word_dim	200	Token embedding dimension
Word_LSTM_dim	100	Token size in LSTM hidden layer
Word_bidirectional	TRUE	Using Bi-LSTM
Word Embedding	TRUE	Using word embedding
CRF	TRUE	Using CRF
Dropout	1	Input droupout
Learning method	SGD	SGD Adadelta Adam
Abbreviation	TRUE	Using Ab3P

In addition, the increase of the hidden layer dimension of Bi-LSTM network may lead to high computational complexity. To optimize the network layers, we have tried different dimensions of hidden layers ranging from 50 to 200 incrementally, with a step of 50, to test the performance of the Bi-LSTM network on the training dataset. From the result shown in Table 4, the F1 score is 0.768 using 50 dimensions of hidden layers and is increased to 0.802 using 100 dimensions of hidden layers. However, the F1 score drops to 0.753 and 0.768 when the dimension number of the hidden layers is increased to 150 and 200, respectively. In order to have a lower computational complexity, we select 100 as the best dimension number of hidden layers for the Bi-LSTM network.

**Table 4:** Effects of dimension settings of hidden layer dimension in Bi-LSTM

	Dimensions	Precision	Recall	F1
Bi-LSTM	50	0.802	0.738	0.768
	**100**	**0.848**	**0.761**	**0.802**
	150	0.838	0.684	0.753
	200	0.848	0.702	0.768

The highest values are denoted in bold type

The number of word embedding dimensions may also affect the method performance and computational complexity. Similarly, we set the word embedding dimensions from 50 to 200, with a step of 50. From the result shown in the Table 5, the highest F1 score is 0.862 when the dimension equals to 200. Consequently, we use 200, which is also commonly used in many other NER tasks as the best dimension setting in word embedding generation.

**Table 5:** Effects of different parameter settings of word embedding dimensions

	Dimensions	Precision	Recall	F1
Word embeddings	50	0.816	0.737	0.774
	100	0.834	0.750	0.790
	150	0.859	0.686	0.763
	**200**	**0.866**	**0.858**	**0.862**

The highest values are denoted in bold type

### Results

During word embedding training, different training data sources may affect the quality of generated word embedding. We use three datasets: 1) A PubMed dataset composed of 22,120,000 paper abstracts. 2) A PMC dataset containing 672,000 full-text publications, and 3) A Wikipedia dataset containing 3,750,000 articles.

We test the performance of disease NER using different combinations of the datasets. As shown in Table 6, with respect to F1 score, using the PubMed (abstract) and the PMC (full text) separately achieve an F1 score of 0.843 and 0.861, respectively. Using the PubMed (abstract) + PMC (full text) obtains the best F1 performance.

**Table 6:** Performance comparison using different combinations of external training datasets

Pre-Data Sets	Precision	Recall	F1
Wikipedia	0.842	0.838	0.840
PMC (full text)	0.866	0.856	0.861
PubMed (abstract)	0.847	0.838	0.843
PubMed (abstract) + PMC (full text)	**0.866**	**0.858**	**0.862**
Wikipedia+PubMed (abstract) + PMC (full text)	0.865	0.858	0.861

The highest values are denoted in bold type

From the result, Wikipedia is not effective on both independent usage and combination. This might be caused by our incomplete Wikipedia training dataset, since the dataset contained only part of disease named entries and some disease names were not being covered. Moreover, Wikipedia is not a specialized medical corpus thus much non-medical content were involved. The reason was also reported by [36] similarly. We therefore use the combination of the PubMed (abstract) and the PMC (full text) as the external datasets for word embedding pre-training.

In order to verify the robustness of the proposed SBLC model, we evaluate the performance using different sizes of the test dataset increasing from 10 to 100 abstracts with a step of 10. We apply a bootstrap sampling method on the test data set using put-back sampling method for 100 times. After that, we assess the statistical significance of F1 scores by computing confidence intervals at the 95% level. In each round, five different strategies by setting different SBLC parameters are used for comparison. As mentioned above, SBLC was the method with the full functions; SBLC(− semantic word embedding) represented SBLC without semantic word embedding layer; SBLC(− word embedding) represents the SBLC without word embedding in the training process; SBLC(− Bi-LSTM) denoted SBLC without Bi-LSTM network; and SBLC(− CRF) denoted the SBLC without the CRF layer.

**Fig. 2:**
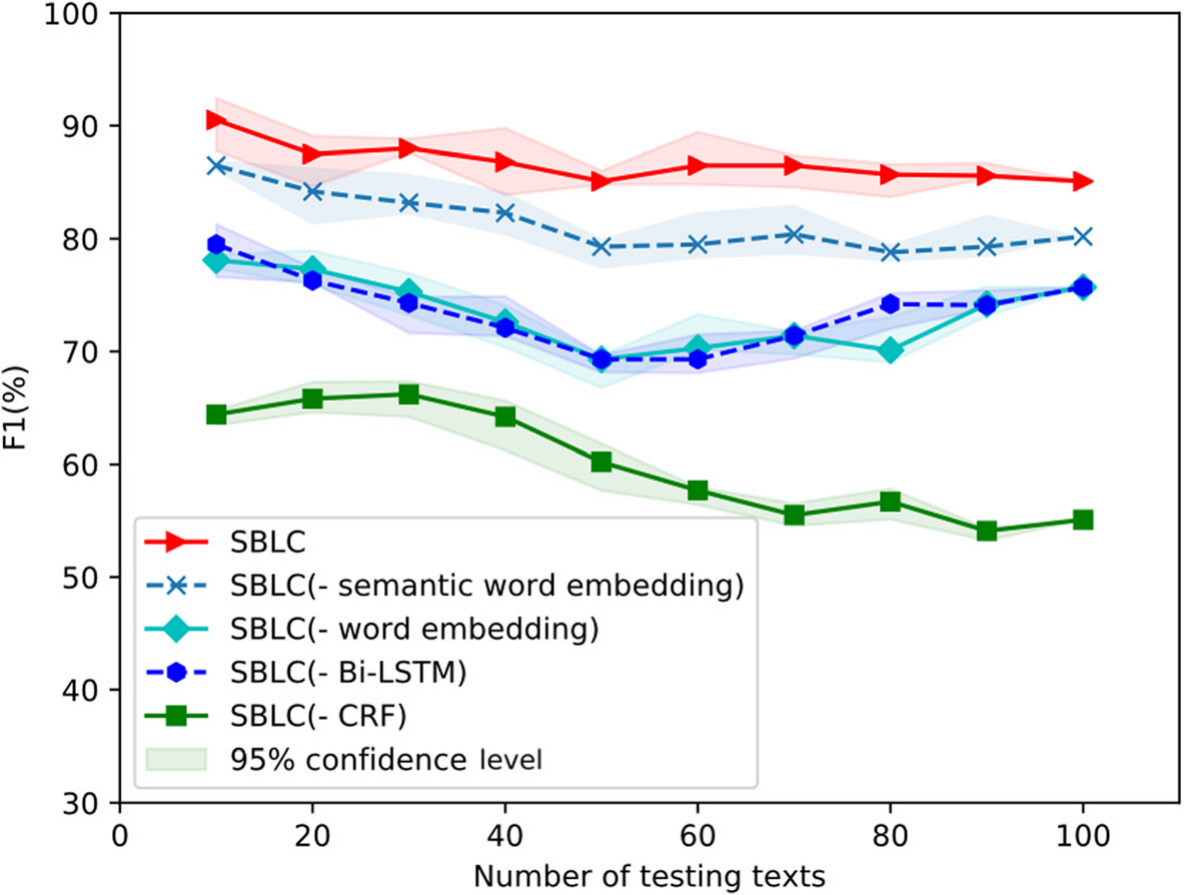
The performance of SBLC using different numbers of testing texts. The lines are the averaged F1 for 100 times testing and the shaded areas are at the 95% confidence level

Without Bi-LSTM, the model acquires the widest range of variability and poor robustness. It shows that Bi-LSTM contributes a lot to the robustness of the SBLC model. The performances of the models without semantic word embedding nor word embedding are close to each other. The robustness of the SLBC model is generally smoother, compared to the two methods. The F1 scores using different numbers of testing texts are shown in Fig. 2.

In addition, we test the performance of SBLC by comparing it with different strategies considering contributions from four parts: Ab3p, CRF, Bi-LSTM, Word Embedding. The comparison results are shown in Table 7. CRF uses the CRF layer structure only for NER. The precision, recall, F1 score is 0.701, 0.675 and 0.688. Bi-LSTM uses the Bi-LSTM layer structure only. The precision, recall, F1 score is 0.600, 0.425 and 0.498. While adding Ab3p on the basis of CRF, Ab3p + CRF obtains a precision and a recall of 0.726 and 0.689, respectively. By adding abbreviations on the basis of Bi-LSTM, Ab3p + Bi-LSTM obtains a precision and a recall of 0.645 and 0.452, respectively. Utilizing both CRF and Bi-LSTM layers, Bi-LSTM + CRF achieves a precision, a recall, and an F1 score of 0.806, 0.800 and 0.803, which improves the overall performance. Combining Ab3p, Bi-LSTM and CRF layers, Ab3p + Bi-LSTM + CRF improves the precision, recall, and F1 score to 0.813, 0.808 and 0.811. Combining Word Embedding and Bi-LSTM layers, Word Embedding + Bi-LSTM achieves a precision, a recall, and an F1 score of 0.675, 0.501 and 0.575. Word Embedding + CRF obtains a precision, a recall, and an F1 score of 0.821, 0.772 and 0.796. Combining Word Embedding, Bi-LSTM and CRF layers, Word Embedding + Bi-LSTM + CRF obtains a precision, a recall, and an F1 score of 0.842, 0.828 and 0.835. Ab3p + Word Embedding + Bi-LSTM, by combining Ab3p, Word Embedding and Bi-LSTM layers, obtains a precision, a recall, and an F1 score of 0.613, 0.689 and 0.648. Combining Ab3p, Word Embedding and CRF layers, Ab3p + Word Embedding + CRF obtains a precision, a recall, and an F1 score of 0.846, 0.786 and 0.815. Ab3p + Word Embedding + Bi-LSTM + CRF (SBLC) obtains the highest precision, recall, and F1 score of 0.866, 0.858 and 0.862.

**Table 7:** Effects of different parameter settings and the final optimized result

Parameter	Precision	Recall	F1
CRF	0.701	0.675	0.688
Bi-LSTM	0.600	0.425	0.498
Ab3p + CRF	0.726	0.689	0.707
Ab3p + Bi-LSTM	0.645	0.452	0.532
Bi-LSTM + CRF	0.806	0.800	0.803
Ab3p + Bi-LSTM + CRF	0.813	0.808	0.811
Word Embedding + Bi-LSTM	0.675	0.501	0.575
Word Embedding + CRF	0.821	0.772	0.796
Word Embedding + Bi-LSTM + CRF	0.842	0.828	0.835
Ab3p + Word Embedding + Bi-LSTM	0.613	0.689	0.648
Ab3p + Word Embedding + CRF	0.846	0.786	0.815
Ab3p + Word Embedding + Bi-LSTM + CRF (SBLC)	**0.866**	**0.858**	**0.862**

The highest values are denoted in bold type

The fourth experiment compares the performances of the proposed SBLC model with those of the above mentioned 9 baseline methods. For MetaMap, we further consider the usage of two filtering strategies: semantic type filtering and MEDIC filtering. For TaggerOne, we further use normalization leveraging external resource. Comparison results are shown in Table 8. The widely-used cTAKES obtain an F1 score of 0.506 and the MetaMap increased the F1 score to 0.559. The inference method acquires an F1 score of 0.637. The three combinations of CRF strategies CRF + CMT, CRF + MeSH and CRF + UMLS obtain F1 scores of 0.735, 0.746 and 0.756. The state-of-the-art methods DNorm and TaggerOne, both developed by NIH, achieve relatively higher F1 scores as 0.798 and 0.829, respectively. The deep learning-based method C-LSTM-CRF obtains an F1 of 0.802, while the recent DNER has an F1 score of 0.843. Our SBLC achieves the highest F1 score of 0.862, outperforming all the baseline methods. The comparison results show the effectiveness of our proposed SBLC method.

**Table 8:** The performance comparison of our SBLC model with the baseline methods on the same NCBI test dataset

Methods	Precision	Recall	F1
Dictionary look-up [2]	0.213	0.718	0.316
cTAKES (version 4.0) [15]	0.476	0.541	0.506
MetaMap (semantic type filtering) [14]	0.495	0.679	0.541
MetaMap (MEDIC filtering) [14]	0.510	0.702	0.559
Inference method [2]	0.597	0.731	0.637
CRF + CMT [34]	0.795	0.683	0.735
CRF + MeSH [34]	0.855	0.660	0.746
CRF + UMLS [34]	0.839	0.688	0.756
DNorm [3]	0.822	0.775	0.798
C-Bi-LSTM-CRF [34]	0.848	0.761	0.802
TaggerOne [22]	0.835	0.796	0.815
TaggerOne(+ normalization) [22]	0.851	0.808	0.829
DNER [24]	0.853	0.833	0.843
SBLC	**0.866**	**0.858**	**0.862**

The highest values are denoted in bold type

## Discussion

### Error analysis

We analyze all the error cases from our SBLC method, and summarize the error cases as the following three types.

1) The complex compound words cause difficulties in disease NER. For example, the disease name “insulin-dependent diabetes mellitus” (MeSH ID D003922) has a joint mark “-” but SBLC can recognize “diabetes mellitus” only. This might be due to the insufficient amount of training data, which cause the incorrect identification of complex disease named entities and compound words.

2) Long disease mentions might cause NER failures. For example, “demyelination of the cerebral white matter” (D003711) and “disorder of glycoprotein metabolism” (DiseaseClass, D008661) are two long disease names failed to be recognized by SBLC. We further identify the length of these error cases with long disease names, and find that the unidentified disease names usually contain more than 3 words. This is a challenge for disease NER, particularly with the appearance of more and more disease names.

3) Some rare disease names appear in the testing dataset only. For example, Non-Hodgkins lymphoma (D008228) is not appeared in the training dataset, thus it is missed in the NER on the testing dataset.

### Medical semantic word embedding

**Fig. 3:**
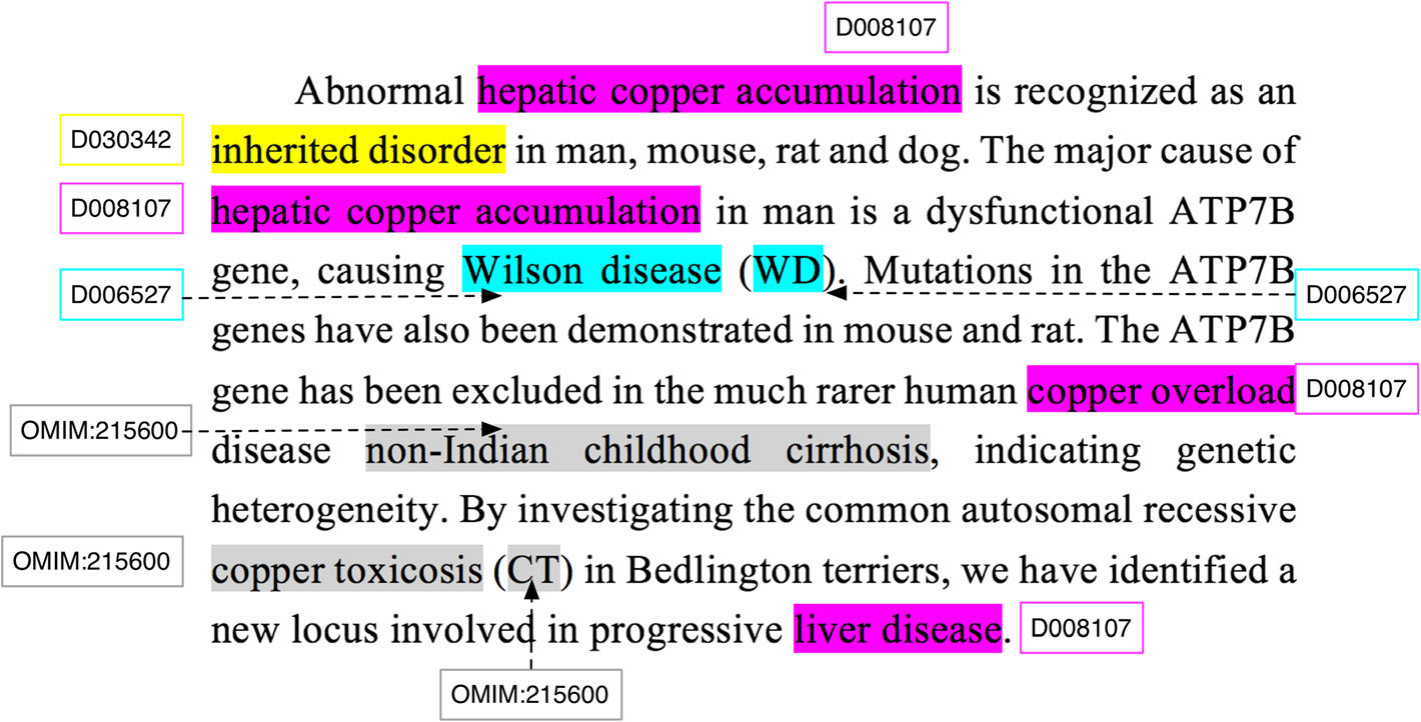
The annotations of the identified disease named entities

In a medical NER task, word is a fundamental unit and word semantics is proved to be useful. The trained semantics could be further enhanced as a feature for higher-level neural network training. For example, the disease NER result on a PubMed article (PID 9949209) in the testing dataset is shown in Fig. 3. The words with colored background in purple, blue, gray and yellow denote the four identified unique disease mentions. These mentions are further normalized to standard concepts marked with associated rectangle boxes containing unique concept id.

**Fig. 4:**
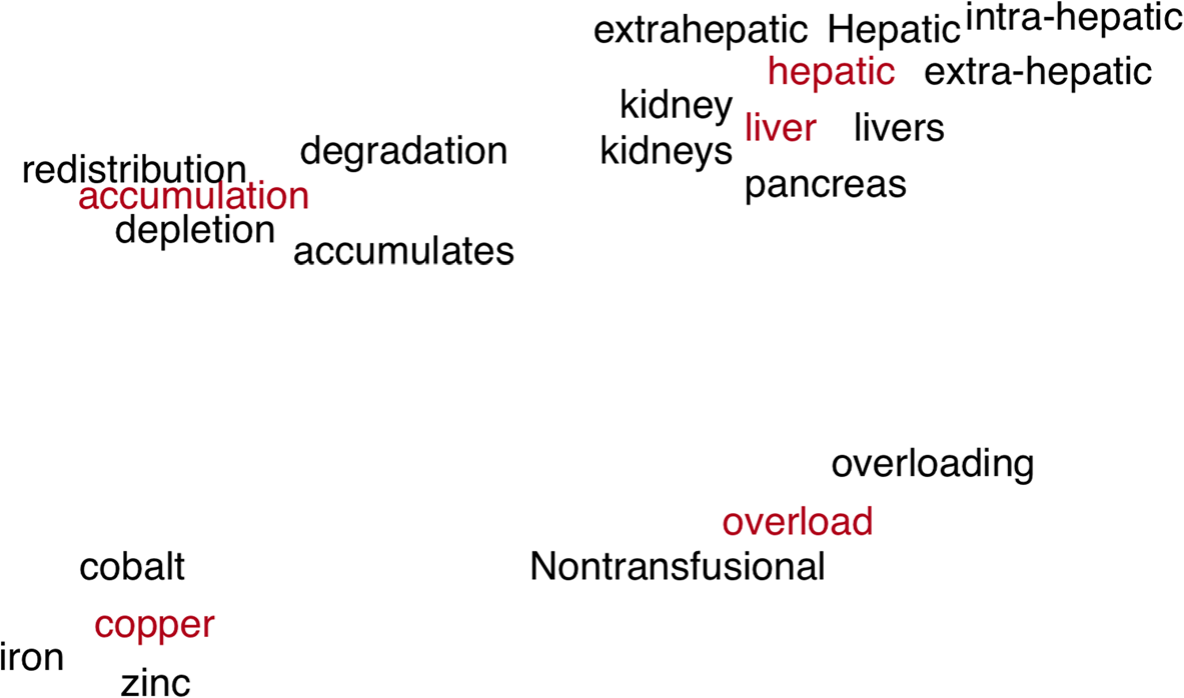
The example word embedding projected to a two-dimensional space

In SBLC, NEG skip-gram is used to train word embeddings and the trained embeddings could reflect the semantic distances among the learned disease concepts. For example, based on the same example above, SBLC calculates the similarities among all the identified disease concepts using the Cosine similarity measure. The results are reported in Table 9. Words in different capitalization and tense, or synonymy are identified and assigned with a similarity weights. In order to view the similarity among the identified disease concepts, we map the concepts to a two-dimensional space, as shown in Fig. 4. The closer the words, the more semantically similar they become. For example, the closest semantics to the word “liver” are “kidney”, “hepatic”, “pancreas”, “kidneys”, and “livers”.

**Table 9:** The semantic similarity among the identified disease concepts using Cosine similarity measure

hepatic		copper		accumulation		overload		liver	
Hepatic	0.784	cobalt	0.849	depletion	0.736	overloading	0.807	kidney	0.81
liver	0.770	nickel	0.831	accumulates	0.688	Nontransfusional	0.672	hepatic	0.77
extra-hepatic	0.738	manganese	0.824	overaccumulation	0.684	overload-related	0.632	pancreas	0.741
intra-hepatic	0.733	iron	0.811	degradation	0.683	overload-induced	0.626	kidneys	0.716
extrahepatic	0.714	zinc	0.799	redistribution	0.681	dyshomeostasis	0.611	livers	0.698

## Conclusions

In this paper, we proposed a new deep learning-based model named as SBLC. The model utilized semantic word embeddings, bidirectional LSTM, CRF, and Ab3P. Based on a standard NCBI disease dataset, we compared the SBLC with 9 state-of-the-art methods including MetaMap, cTAKES, DNorm, and TaggerOne. The results showed that the SBLC model achieved the best performance, indicating the effectiveness of SBLC in disease named entity recognition.

## Abbreviations

Bi-LSTM: Bidirectional Long Short Term Memory networks
CMT: Convergent Medical Terminology
CRF: Conditional Random Fields
NER: Named Entity Recognition
UMLS: Unified Medical Language System
